# Normative data and discriminative properties of short form 36 (SF-36) in Turkish urban population

**DOI:** 10.1186/1471-2458-6-247

**Published:** 2006-10-09

**Authors:** Yucel Demiral, Gul Ergor, Belgin Unal, Semih Semin, Yildiz Akvardar, Berna Kıvırcık, Köksal Alptekin

**Affiliations:** 1 Department of Public Health, Dokuz Eylul University School of Medicine, Izmir, Turkey; 2 Department of Medical Ethics, Dokuz Eylul University School of Medicine, Izmir, Turkey; 3 Department of Psychiatry, Dokuz Eylul University School of Medicine, Izmir, Turkey

## Abstract

**Background:**

SF-36 has been both translated into different languages and adapted to different cultures to obtain comparable data on health status internationally. However there have been only a limited number of studies focused on the discriminative ability of SF-36 regarding social and disease status in developing countries. The aim of this study was to obtain population norms of the short form 36 (SF-36) health survey and the association of SF-36 domains with demographic and socioeconomic variables in an urban population in Turkey.

**Methods:**

A cross-sectional study. Face to face interviews were carried out with a sample of households. The sample was systematically selected from two urban Health Districts in Izmir, Turkey. The study group consisted of 1,279 people selected from a study population of 46,290 people aged 18 and over.

**Results:**

Internal consistencies of the scales were high, with the exception of mental health and vitality. Physical health scales were associated with both age and gender. On the other hand, mental health scales were less strongly associated with age and gender. Women reported poorer health compared to men in general. Social risk factors (employment status, lower education and economic strain) were associated with worse health profiles. The SF-36 was found to be capable of discriminating disease status.

**Conclusion:**

Our findings, cautiously generalisable to urban population, suggest that the SF-36 can be a valuable tool for studies on health outcomes in Turkish population. SF-36 may also be a promising measure for research on health inequalities in Turkey and other developing countries.

## Background

The Short Form-36 Health Survey (SF-36) has been constructed to represent eight health concepts[1]. It has been referred to as a generic measure since it assesses health concepts that are pertinent to everyone's functional status and well-being. This generic measure can be used in diseased groups as well as general populations. SF-36 also allows comparisons between different disease groups, i.e. patients with rheumatologic disorders versus cardiac patients. SF-36 has been reported as useful in clinical practice, research, health policy evaluations, and population surveys [1-7].

SF-36 has been both translated into different languages and adapted to different cultures to obtain comparable data on health status internationally. It has been shown as reliable and able to detect differences between groups defined by age, sex, socio-economic status, geographical region and clinical conditions [2,8]. One of the well-documented cross-cultural adapting studies was performed for SF-36 by the International Quality of Life Assessment Project (IQOLA). In this project the researchers highlighted the importance of cultural appropriateness, yet most of the countries included in the Project were developed ones [6,9,10]. It has been shown that SF-36 is sensitive to social factors such as social class and disease status in population surveys and primary care settings [11,12]. Specific relationships between social determinants such as economical, employment and educational status and health status may be observed by SF36. For instance, people in better social positions are expected to report better health status than the lower social groups. Similarly people with a disease are more likely to have lower SF36 scores than people without a disease. These hypotheses have been revealed in number of studies from developed countries. On the other hand, to date, only a limited number of studies have focused on the discriminative ability of SF-36 regarding social and disease status in developing countries [13].

SF-36 was translated in to Turkish and validation studies of Turkish version of SF-36 were carried out in patient groups in 1999 and 2005[14,15]. In the present study we aimed to obtain population norms for the Turkish version of SF-36. We also aimed to ascertain the association of SF-36 domains with demographic and socioeconomic variables and self-reported ill-health in a general urban population.

## Methods

### Sampling and data collection

The study population consisted of 46,290 people aged 18 and over who lived in Narlidere and Balcova Health Districts in Izmir. Izmir is the third largest and economically developed city in Turkey with a population of 2.4 million. This study was a part of a survey which aimed to determine prevalence of psychotic disorders in the adult population. The sample size required for this study was 1,473 (rounded to 1,500) people, assuming 1% prevalence of schizophrenia and 0.5% error and with 95% confidence level. Households were considered as sampling units. The average number of people over 18 years of age for each household was calculated as 2.5 from the two Health Districts' registers. The sample size was estimated as 600 households, which was boosted by 10%, so that 660 households were targeted for the survey. Household lists were obtained from the Narlidere and Balcova Health Districts and houses were selected systematically from these lists. The initial house was selected by generating a random number and then every 28th (sampling interval) household was included in the sample.

We compared the age, sex and educational structure of the sample with Turkish urban population figures obtained from Turkish Statistics Institute. The sex and educational level of the sample was similar with the general urban population of Turkey. We applied Turkish age weights to all SF-36 scores obtained from the sample.

Houses were visited by trained study team members and informed the eligible persons, e.g. older than 18 years of age, about the study. When the eligible person in the household was absent at the time of visit, two more consecutive visits were scheduled. In order to obtain the data, SF-36 version 1.0 and sociodemographic information questionnaires were completed using a face-to-face interview technique after asking verbal consent from the participants. The study included a total of 1,279 participants. The study was approved by Clinical Research Ethics Committee of Dokuz Eylul University School of Medicine.

### Questionnaires and scaling

The SF-36 version 1.0 is a short form questionnaire with 36 items that measure eight health related quality of life domains: physical functioning (PF), social functioning (SF), role limitation due to physical problems (RP), role limitation due to emotional problems (RE), mental health (MH), energy and vitality (VT), bodily pain (BP), and general perception of health (GH). The SF-36 also includes an item to assess changes in respondent's health status during the past year [16,17]. For each quality of life domain tested, item scores were coded, summed, and transformed into a scale from 0 (worst) to 100 (best) using the standard SF-36 scoring algorithms [1]. Physical and mental summary component scale (PCS and MCS respectively) scores were also calculated using algorithm described by the developers[18]. We calculated the PCS and MCS by using both standard (i.e. United States weights) and country-specific algorithms (i.e. Turkish weights), in order to interpret summary scales comprehensively.

The eight scales of SF-36 were standardized using a *z*-score transformation with the mean and standard deviations obtained from our sample. After obtaining *z*-scores for each scale, the aggregate scores for the physical and mental component scale scores were calculated. The country specific PCS and MCS were computed by multiplying each scale's *z*-score by its respective factor weights based on the Turkish sample. Finally, these scores were standardised to a T-score, where the mean was set to 50 and the standard deviation was 10.

A second questionnaire was used to obtain socio-economic and demographic data. Chronic illness was assessed by 12 specific disease questions (yes-no form), i.e. does the participant suffer from or have they suffered from diabetes, hypertension, myocardial infarction, asthma, tuberculosis, cancer, stroke, epilepsy, arthritis, a disability, depression, and one open ended question coded as "other". The disease status was further dichotomised as "none" or "any".

Employment status and perceived economic position were used to evaluate economic status. Any person who had paid work was considered as employed and the others unemployed. Perceived economic position was evaluated by a single question scaled using a four point Gutman scale. The question was "how do you consider your economic status?" and the responses were "good, fair, bad and very bad". "Good and fair" were regrouped against "bad and very bad".

### Statistical analyses

Eight domains and two summary scale scores of the SF-36 were calculated [1]. Population norms were standardised according to Turkish urban population age structure. Internal reliability of the SF-36 was assessed using Cronbach's alpha coefficient. Floor and ceiling effects were expressed as the percentages of bottom and top scores of the scales, respectively. Principal component factor analysis (PCA) was used to obtain factorial structure of the SF-36 domains. When performing PCA the number of factors defined by eigenvalues ≥1.0.

Criterion validity was examined by comparing scores of groups categorised on the basis of disease status. Mann-Whitney U and Kruskal-Wallis tests were used to compare group differences, since the distribution of eight domains was skewed.

Logistic regression analysis was used to assess discriminative properties of SF36 for socioeconomic variables and disease status. In the logistic regression models SF-36 scores were included as continuous variables. Firstly, because there is no universally accepted cut-off levels for SF-36 scale scores. Secondly, visually graphs between SF-36 scale scores and sociodemographic variables showed quite linear trends.

Age and sex adjusted odds ratios (OR) and 95% confidence intervals (CI) for one unit increase in SF36 domains for economical, employment, educational and disease status were estimated from the logistic regression models. Unemployment was defined as people who are not employed and seeking jobs for the logistic regression analysis. SPSS Version 11.0 was used for all statistical analyses.

## Results

1,279 people completed the study, yielding response rates of 87% for houses (575 out of 660); and 83% for eligible people, i.e. 18 and over years old (1,279 out of 1,551). Sixteen people refused to join the study and 37 could not be reached after three consecutive visits. One questionnaire had more than half missing items in SF-36 and was excluded from the study.

The mean age of the study group was 42.9 ± 14.7 and 47.6% were men. Nine percent of the study group was over 65. There were no significant differences in age groups by gender. Most of the participants rated their economical position as "fair" (81.2%). Seven percent of the total study sample had no formal education (Table 1). Unemployed participants consisted of 4.1% of the study population.

Normative values and internal consistency reliability coefficients for each scale are presented in Table 2. High ceiling effects were obtained for the PF, RP, SF and RE scales, with 71%, 88%, 83% and 93% of respondents obtaining the highest possible scale score, respectively. In addition, the median value was equal to the maximum possible score of 100 for five of the eight scales. Ceiling effects were not observed for the GH, VT and MH scales.

VT and MH scales have a low level of internal consistency, with their coefficients being 0.65 and 0.64, respectively. Three of the coefficients were above the generally accepted value of 0.90 for individual comparisons, namely PF (0.98), RP (0.97) and RE (0.93).

Results from two participants showed negative values for the physical component summary scale (PCS). PCS values ranged from – 1.1 to 63.0. Mental component summary scale (MCS) values ranged between 0.1 and 78.0. Median values were 54 and 52 for PCS and MCS, respectively (Table 2).

Women reported poorer health compared to men for all quality of life variables except MH, V and MCS (Table 3).

Mean (SD) values for SF-36 variables by age groups are presented in Table 4. The highest values for all variables were observed in the 18 to 44 age group. A significant decreasing gradient was found between age groups for all except MCS and MH scores. Post- hoc analysis of age groups by Mann-Whitney U tests revealed that the results from participants in the 65 and over age groups differed significantly from those seen in their younger counterparts.

The results of the varimax rotation solution for Turkish SF-36 and US loads were presented in Table 5. Two factors were derived from the subscales. Mean PCS and MCS scores obtained by using Turkish and US algorithms were also presented in Table 5. The total variability explained by the two factors was %65. Factor one was clearly associated with PCS. SF and RE were clustered in factor 1. VT was clustered in factor 2. There were statistically significant differences for both PCS and MCS scores obtained using Turkish and US algorithms (p < 0.0001). The correlations of two types of calculation methods were 0.96 and 0.92 for PCS and MCS respectively (correlation data were not shown).

All SF-36 domains showed significant associations with economical position, employment status, educational and disease status (Table 6).

In this study 36.2% (n = 463) of the study group declared that they currently had one of the physician-diagnosed diseases from the 12 items diseases list. Those participants who had reported a disease had lower scores for all variables; and these differences were significant except for MCS (Table 6).

## Discussion

This is the largest study in Turkey which aimed to obtain population norms and discriminative power for the SF-36 health survey questionnaire in a community sample.

The field study was performed fastidiously and thus resulted in a good response rate. We used face-to-face interviews for data collection, which increased data quality of the SF-36 questionnaire (only one person was excluded because of missing data). On the other hand the mode of administration of the questionnaire may have an influence on how people report on or rate their health status. To minimize this limitation we have trained and standardized our interviewers on administering the questionnaire.

The sample size of our study might be slightly smaller than suggested by Gandek et al [19] for studies aiming to determine population norms of SF-36. However, our sample size enabled us to detect minimum score differences (i.e. effect size = 0.2) with alpha = 0.05 and power = 80% [20]. Furthermore, our study group is a representative sample of the general, healthy, urban population, since we used systematic sampling method. We have avoided over analysing the data and presented the population norms of SF-36 and the association of SF-36 domains with demographic and socioeconomic variables only.

### Internal consistency

Internal consistencies of the scales were high for six domains (over 0.80). Two domains, namely vitality and mental health, had low internal consistency coefficients. In a recent study, higher Cronbach's α values were obtained for VT and MH among the Turkish cancer patients (0.87, 0.82, respectively). In our study, lower Cronbach's α for VT and MH subscales suggest that either more refinement is needed before full cultural adaptation, or that the differences reflect the diversity of our study sample compared to Pinar's study [14]. Low internal consistency coefficients for these domains were reported previously in Chinese Americans [21]. That study indicated that the SF-36 met minimum psychometric criteria with the exception of vitality and mental health. Similarly, Ahmed et al reported a failure in internal consistency for the vitality scale in a Bangladesh version of SF-36 [22]. In our study, the high level of internal consistency in the PF, RP and RE domains were somewhat unusual. The Cronbach's alpha= 0.90 is the generally accepted value for individual comparisons. The accepted value for group comparisons is 0.70 [3]. We think that Cronbach's alpha coefficients being over 0.90 was not due to the fact that these items do not apply to Turkish lifestyle. Rather, it is the result of our sample being a young and relatively healthy people since only 9% of our sample is over the age of 65 and even in this group the majority are capable of performing these daily living activities.

### Normative data

The results of our study provide normative data for SF-36 for a Turkish urban population. Considerable ceiling effects were obtained for five out of eight scales that had the median score of 100 (perfect score). Previous studies have also reported identical skewed distribution of the SF-36 scales in patients and healthy people [7,13,21]. Possible explanations for this finding could be differences in translation and/or interpretation of the item and response choice contents, or cultural norms that may favour socially desirable response choices that reflect better health.

Turkish population norms were higher than the United States general population norms with the exception of mental health [1]. This may reflect the general distress of the population since the data were collected in year 2002, just after a major economic crisis in Turkey. On the other hand 90.3% of the study group rated their health status as the same or better compared to the previous year. Since there is no logical reason an economic crisis would directly cause poor physical health (except where it was somatized), the lower mental health scores may reflect a general anxiety factor related to worry about maintaining economic wellbeing.

In general, the mean score for social functioning obtained from our study was higher than the industrialised countries [1,10,12,23,24]. Interestingly, previous data from some other developing countries indicated the same trend [13,22,25], i.e. higher scores were obtained for SF. It could be speculated that social relations and collectivism in developing countries might play a pivotal role in individual's daily life. Thus, SF is affected by physical or mental ill health to a lesser extent than in western societies. This may also reflect the differences in the meaning of SF items regarding the expectations of life between eastern and western cultures.

### Summary scale scores

Comparing PCS and MCS has several advantages. Summary measures reduce the number of statistical requirements and provide a useful interpretation with a little loss of information [26]. This dual component model of SF-36 was first described in the United States and then replicated for some other western countries by using their own general population data. In theory, country specific scores have the advantage of better representing the structure of health in each country. However, country specific scores have the disadvantage of precluding comparisons between countries [26]. We have calculated the PCS and MCS by using both standard and country specific algorithms to interpret summary scales of the SF-36 comprehensively [26,27]. Results from our study revealed that there was a significant difference between the scores when different algorithms were used. Factor loads for SF and RE were showed higher specificity for PCS. Turkish SF-36 factor loads were comparable with the US factor loads except RE. On the other hand, loads for SF and RE domains were 0.31 and 0.42 respectively for MCS and considered as "shared high loadings"[28]. However, the country specific scores should be treated with caution when used for international comparisons.

### Discriminative power

Our findings on the discriminative power of SF-36 were similar to the previous studies [2,6,10,13,29,30]. Regarding the physical and mental components, SF-36 revealed a clear discrimination between diseased and non-diseased groups, suggesting good construct validity [11,24]. Usually, women report worse scores than men in most SF-36 scales, including mental health. In our study, men were found to be significantly healthier than women in general. However there was not significant difference between men and women. This might be result of relatively better social status of women in the study group. On the other hand the validity of MCS of the Turkish version of SF-36 might be questionable, especially when relatively lower factor loads for SF and RE which are used to estimate MCS. Health was worse in older aged groups. It has always been a challenge to demonstrate the disparities in health status according to social class determinants. In this study, it was shown that SF-36 was capable of detecting the differences of health status among the social variables such as economical status, employment status, and educational level. When accounting for disease oriented outcome measures such as cardiovascular morbidity or mortality, there has been substantial evidence that the effect of social class differences on health in developing countries is unexpectedly contrary to the effect in industrialised countries, i.e. the higher the class, the higher the risk of morbidity [31]. One reason for this discrepancy may be that social classes in developing countries are not as well structured as those in developed ones and thus result in a failure to show an association between social classes and health. Secondly, culture or behaviour based social variables might play a more dominant role in developing countries than in developed countries. Thirdly, higher social classes in the developing countries have the wealth to afford lifestyles characteristic of those seen in developed countries, such as a high fat diet and sedentary living.

### Strengths and limitations of this study

Our study is the first population based study that aimed to provide population norms of SF-36 in Turkey. However our results could not be generalisable to whole Turkish population. Because, our study sample was consisted of only urban population in a single city, Izmir. Although our sample resembles the general urban population of Turkey in terms of age, sex and education level, it may still differ from the rural population of other cities in Turkey. Apparently, SF-36 items may not be generalisable to Turkish rural population.

Our findings were promising for research on inequalities in health in Turkey since they showed a clear association between primary social variables (economic status, employment and education level) and health. This finding may also be valid to other developing countries.

## Conclusion

With its limited generalisability discussed in previous paragraph, our study provided population norms of SF-36 that could be used for comparisons across different settings in a Turkish population. Moreover, the discriminative power of SF-36 in research on health inequalities may be of great importance in Turkey and other developing countries.

## Competing interests

The author(s) declare that they have no competing interests.

## Authors' contributions

YD designed the study, organised the data collection and performed the data analysis; he drafted and revised the manuscript. GE participated in the initial study design and revision of the article. BU participated in data analysis and revision of the manuscript. SS participated in the initial study design, collection of data and revision of the manuscript. YA, BK and KA participated in the initial study design, and revising the manuscript. All authors read and approved the final version of this manuscript.

## Pre-publication history

The pre-publication history for this paper can be accessed here:



## Figures and Tables

**Table 1:** Descriptive statistics of the study sample (N = 1279) and Turkey

	Turkey %	Study sample %
**Age groups **		
18–24	-	12.0
25–34	27	20.3
35–44	22	23.8
45–54	15	22.0
55–64	10	12.8
65+	10	9.1
**Sex **		
Male	50	47.6
**Educational level**		
No formal education	10	7.0
Primary	34	30.4
Elementary	12	13.0
High school	23	25.0
University	21	24.6
**Economic position **	**NA **	
Good		9.7
Fair		81.2
Bad, very bad		9.1
**Employment status **	**NA **	
White collar		16.2
Blue collar		19.0
Self employed		3.8
Unemployed		4.1
Retired		17.0
Housewife		34.7
Student	-	5.2

NA: not available

**Table 2:** SF-36 scale results for the study group

**Variables (N) **	**Mean (SD) **	**Median **	**Floor (%) **	**Ceiling (%) **	**Cronbach's alpha **
PF (1279)	83.8 ± 20.0	100	0 (2.8)	100 (70.7)	0.98
RP (1279)	86.3 ± 24.9	100	0 (9.0)	100 (87.6)	0.97
BP (1279)	82.9 ± 18.9	100	0 (0.3)	100 (61.5)	0.88
GH (1279)	71.6 ± 16.1	72	0 (0.1)	100 (5.6)	0.84
VT (1271)	64.5 ± 12.9	70	0 (0.1)	100 (0.7)	0.65
SF (1279)	91.0 ± 12.9	100	0 (0.2)	100 (83.4)	0.81
RE (1279)	90.1 ± 19.4	100	0 (3.9)	100 (93.1)	0.93
MH (1271)	71.0 ± 11.0	76	0 (0.0)	100 (0.9)	0.64

PCS* (1271)	47.9 ± 8.9	54	-1.1 (0.1)	63.0 (0.1)	

MCS* (1271)	47.7 ± 9.4	52	0.1 (0.1)	78.1 (0.1)	

* Country specific algorithms

**Table 3:** Mean (SD) scores for eight variables of SF-36 for women and men

**Variables (N) **	**Women (n = 670)** **Mean ± SD **	**Men (n = 609)** **Mean ± SD **	P*
PF (1279)	80.6 ± 21.7	87.2 ± 17.1	0.000
RP (1279)	82.9 ± 28.6	89.8 ± 19.3	0.000
BP (1279)	81.0 ± 20.2	85.1 ± 16.4	0.001
GH (1279)	69.1 ± 16.9	73.6 ± 14.9	0.000
VT(1271)	63.4 ± 13.7	65.7 ± 11.9	0.098
SF (1279)	90.1 ± 12.9	91.7 ± 12.8	0.017
RE (1279)	89.0 ± 22.5	92.8 ± 15.1	0.001
MH (1271)	70.1 ± 11.4	71.0 ± 10.6	0.727

PCS** (1271)	46.6 ± 9.9	49.4 ± 7.4	0.000

MCS** (1271)	47.3 ± 9.8	48.2 ± 9.1	0.439

*Mann-Whitney U test

** Country specific algorithms

**Table 4:** Mean (SD) of SF 36 scores according to age groups

**Age groups (N) **	**PF Mean (SD) **	**RP Mean (SD) **	**BP Mean (SD) **	**GH Mean (SD) **	**VT Mean (SD) **	**SF Mean (SD) **	**RE Mean (SD) **	**MH Mean (SD) **	PCS* Mean (SD)	MCS* Mean (SD)
18–44 (717)	94.7 (16.5)	95.3 (20.3)	90.1 (17.6)	77.3 (15.6)	68.6 (12.8)	96.5 (11.9)	95.8 (18.5)	73.6 (11.0)	52.7 (7.5)	49.9 (9.2)
45–64 (446)	81.3 (25.5)	86.8 (32.3)	83.6 (21.4)	70.8 (18.3)	65.7 (14.1)	94.7 (12.9)	95.0 (20.5)	73.5 (11.5)	48.2 (11.0)	49.5 (10.3)
≥ 65 (116)	56.3 (37.2)	64.0 (46.4)	70.3 (25.9)	62.8 (20.7)	60.9 (15.8)	83.7 (24.4)	86.5 (32.5)	72.0 (14.8)	37.7 (16.3)	48.3 (12.3)
P**	0.000	0.000	0.000	0.000	0.000	0.000	0.000	0.687	0.000	0.850

* Country specific algorithms

**Kruskal – Wallis test

**Table 5:** Factor loads obtained from Turkish and US algorithms and comparison of mean scores of Turkish and US algorithms for PCS and MCS

	Factor 1 (TR)	Factor 1 (US)	Factor 2 (TR)	Factor 2 (US)
PF	0.83	0.88	0.01	0.04
RP	0.84	0.78	0.18	0.30
BP	0.77	0.77	0.15	0.24
MH	0.09	0.12	0.90	0.90
RE	0.53	0.19	0.42	0.81
SF	0.69	0.44	0.31	0.71
GH	0.65	0.68	0.34	0.32
VT	0.24	0.59	0.85	0.57
	PCS (TR) (Mean ± SD)	PCS (US) (Mean ± SD)	MCS (TR) (Mean ± SD)	MCS (US) (Mean ± SD)
	49.8 ± 10.8	52.6 ± 8.8	49.6 ± 9.9	51.7 ± 5.7
p*	<0.0001		<0.0001	

*Wilcoxon signed rank test

**Table 6:** Age and sex adjusted odds ratios of SF-36 domains for social determinants and disease status

	**PF OR (95% CI) **	**RP OR (95% CI) **	**BP OR (95% CI) **	**GH OR (95% CI) **	**VT OR (95% CI) **	**SF OR (95% CI) **	**RE OR (95% CI) **	**MH OR (95% CI) **	**PCS OR (95% CI) **	**MCS OR (95% CI) **
**Economical status **										
Fair/good vs Bad/very bad	1.015 (1.008–1.023)	1.016 (1.011–1.021)	1.029 (1.020–1.037)	1.059 (1.047–1.070)	1.069 (1.054–1.084)	1.027 (1.017–1.037)	1.026 (1.020–1.032)	1.089 (1.071–1.106)	1.044 (1.027–1.061)	1.113 (1.091–1.135)
**Employment status **										
Employed vs unemployed	1.012 (0.998–1.026)	1.014 (1.004–1.024)	1.026 (1.013–1.040)	1.027 (1.010–1.044)	1.031 (1.012–1.051)	1.012 (0.994–1.030)	1.010 (0.998–1.022)	1.054 (1.032–1.077)	1.034 (1.005–1.065)	1.058 (1.031–1.086)
**Educational status **										
Secondary or high vs primary	1.006 (1.001–1.012)	1.003 (0.999–1.007)	1.017 (1.011–1.024)	1.011 (1.004–1.018)	1.018 (1.008–1.027)	1.002 (0.993–1.011)	1.005 (0.999–1.011)	1.024 (1.014–1.035)	1.013 (1.001–1.025)	1.025 (1.012–1.037)
**Disease status **										
No vs yes	1.023 (1.017–1.019)	1.022 (1.017–1.028)	1.035 (1.028–1.042)	1.041 (1.033–1.050)	1.015 (1.005–1.024)	1.028 (1.018–1.038)	1.019 (1.012–1.026)	1.018 (1.007–1.029)	1.084 (1.066–1.101)	1.018 (1.006–1.031)

**Country specific algorithms
